# Isolation and characterization of the mink interferon-epsilon gene and its antiviral activity

**DOI:** 10.3389/fvets.2022.972433

**Published:** 2023-01-27

**Authors:** Hailing Zhang, Dongliang Zhang, Shasha Zhang, Hao Liu, Han Wang, Cong Wang, Deying Zou, Bo Hu, Shizhen Lian, Shiying Lu, Xue Bai

**Affiliations:** ^1^Key Laboratory of Special Animal Epidemic Disease, Ministry of Agriculture, Institute of Special Animal and Plant Sciences, Chinese Academy of Agricultural Sciences, Changchun, China; ^2^Key Laboratory of Zoonosis, Ministry of Education, Institute of Zoonosis, College of Animal Science and Veterinary Medicine, Jilin University, Changchun, China; ^3^School of Life Sciences and Engineering, Foshan University, Foshan, China

**Keywords:** mink, interferon-ε, tissue distribution, antiviral activity, interferon-stimulated gene 15, MX1, 2′-5′ oligoadenylate synthetase 1

## Abstract

The interferon (IFN) response is the first line of defense against viral invasion and thus plays a central role in the regulation of the immune response. IFN-epsilon (IFN-ε) is a newly discovered type I IFN that does not require viral induction, unlike other type I IFNs. IFN-ε is constitutively expressed in epithelial cells and plays an important role in mucosal immunity. In this study, we evaluated the biological activity of the mink-IFN (MiIFN)-ε gene in prokaryotic cells. Quantitative reverse transcription-polymerase chain reaction (qRT-PCR) was used to evaluate IFN-ε expression in different mink tissues. MiIFN-ε was highly expressed in brain, lung, tracheal, kidney, intestinal, bladder, ovarian, and testis tissues. There was no significant difference in MiIFN-ε expression between female and male minks, except in the reproductive system. Expression of the small ubiquitin-like modifier (SUMO3)-MiIFN-ε fusion gene was induced by isopropylβ-d-thiogalactoside, and MiIFN-ε was collected after SUMO-specific protease digestion. We tested the antiviral activity of MiIFN-ε against vesicular stomatitis virus (VSV) in epithelial cells of feline kidney 81 (F81). We used qRT-PCR to analyze the expression of several IFN-stimulated genes (ISGs), including ISG15, 2′-5′ oligoadenylate synthetase (2′-5′OAS1), and myxovirus resistance protein 1 (Mx1). Recombinant IFN-ε induced high ISG expression in F81 cells. Compared with those in the cell control group, expressions of ISG15, Mx1, and 2′-5′ OAS1 in the VSV-GFP control, IFN-ε, and MiIFN-ε-inhibited VSV-GFP groups were significantly increased. Compared with those in the VSV-GFP control group, expressions of ISG15 and 2′-5′ OAS1 in the IFN-ε and MiIFN-ε-inhibited VSV-GFP groups were significantly increased, and the differences were highly significant (*p* < 0.0001). IFN-ε played an indirect antiviral role. These findings lay the foundation for detailed investigation of IFN-ε in the future.

## Introduction

Interferons (IFNs) were the first cytokines discovered in vertebrates and have broad antiviral, antitumor, and immunoregulatory activities (1). Ubiquitous in organisms, IFNs are secreted glycoproteins that play a central role in immune response regulation (1). Their antiviral and antitumor properties enable immune prevention and treatment (2–4). According to their gene sequence, chromosome position, and receptor specificity, IFNs are divided into three types: I, II, and III. Type I IFNs include IFN-α, -β, -ω, -ε, -κ, -δ, -ν, -τ, and -ζ (5, 6). Type II IFN comprises an IFN-γ single-gene family, also known as immune IFN (7, 8). Type III comprises IFN-λ, which is similar to type I IFNs (IFN-α and IFN-β) (7, 8).

Type I and III IFNs first bind to the receptor chain IFNAR2 or IFNLR1 with high affinity and then bind to the receptor chain IFNAR10 or IL10Rβ with low affinity to form a signal transduction ternary complex. Receptor dimerization activates JAK1 and TYK2 kinases, resulting in phosphorylation of JAK1 and TYK2 in the cytoplasmic domain of the receptor subunit. This, in turn, triggers the phosphorylation of signal transducers STAT1 and STAT2. The phosphorylated heterodimers of STAT1 and STAT2 form an IFN-stimulated gene factor 3 (ISGF3) complex with IFN regulatory factor 9 (IRF9), which translocates into the nucleus. It binds to IFN-stimulated response elements (ISREs) or gamma-activated sequences (GASs) during the initiation of ISGs (9, 10). The transcription of hundreds of genes involved in the antiviral response is initiated, including ISGs, oligoadenylate synthases (OASs), guanylate-binding proteins (GPBs), nitric oxide synthase 2 (NOS2), and IFN-induced transmembrane proteins (IFITMs). Tripartate motif proteins (TRIMs), including ISG15, Mxs, IFNs, IRFs, and STATs, promote the expression of hundreds of ISGs (11). ISG15, myxovirus resistance protein 1 (Mx1), and 2′, 5′-OAS are major IFN-induced antiviral factors. ISG15 is a precursor protein with a molecular weight of 17 kDa that is cleaved into a mature peptide protein of 15 kDa by protease. The mature ISG15 exposes the LRLRGG motif at the carboxyl terminus and covalently binds to viral proteins and can disrupt their interaction with the host pathway required for replication, disrupt the oligomerization of viral proteins and/or the geometric configuration of the virus, or disrupt the function of viral proteins, resulting in reduced viral replication or changes in the host immune response (12, 13). MxA may inhibit transcription of the virus in the cytoplasm and its subsequent steps. The MxA protein interferes with the cytoplasmic transport of viral mRNA, viral protein synthesis, or translocation of newly generated viral protein to the nucleus. It has been proven to inhibit the replication of influenza A virus, Tokato virus, and vesicular stomatitis virus (14–16). 2′, 5′-Oligoadenylate synthase (2′, 5′-OAS) is a key enzyme in the process of IFN antiviral and anti-cell proliferation. OAS family proteins belong to the template-independent nuclease family. Oligomeric OAS enzymes produce 2′-5′ -linked oligoadenylates (2–5A), which can activate internal ribonuclease RNase L and degrade cell and viral RNA. Thus, RNase L helps to control the early spread of the virus by degrading viral RNA and activating cytoplasmic pattern recognition receptors RIG-I and MDA-5 (17). Therefore, the detection of ISG15, Mx1 and 2′, 5′-OAS could indirectly reflect whether a drug has antiviral effect.

FN-ε is constitutively expressed in epithelial cells of various organs and tissues (18) and has important antiviral and mucosal immune regulatory functions (19). Recombinant human IFN-ε can induce the production of an antiviral protein, MxA, to exhibit antiviral effects and inhibit human immunodeficiency virus replication (20). Recombinant IFN-ε from cattle, camels, dogs, and sheep has also shown significant antiviral activity (21–24). Although IFN-α and IFN-β preparations have good antiviral effects, their long-term use may lead to side effects such as mental anxiety and depression. Gene chip assay results show that IFN-ε can induce various effects favorable for the nervous system (25, 26). Therefore, IFN-ε has great potential for replacing IFN-α and IFN-β and for reducing adverse reactions. Presently, IFN-ε is used for the treatment of viral infectious diseases, such as condyloma acuminatum, hepatitis B and C, and several tumors, including laryngeal multiple papilloma, bladder cancer, and cervical cancer, but these treatments are all in the preclinical research stage. Studies on IFN-ε in animals are lacking; however, the genes and biological activities of IFN-ε have been partially reported in macaques, pigs, cattle, dogs, and mice (27). IFN-ε expression differs among species, and its specific biological functions and detailed mechanisms remain unclear. Previous studies have shown that mink IFN-α significantly inhibits the replication of vesicular stomatitis virus (VSV) and mink enteritis virus (MEV) *in vitro* (28). Whether mink IFN-epsilon has the same antiviral activity is unknown. Here, we describe the construction of a fusion protein, mink IFN-ε, with the SUMO tag, its co-expression with the chaperone protein Tf16 in Escherichia coli, and its purification and antiviral activity.

## Materials and methods

### Cells, viruses, and animals

Feline kidney 81 (F81) cells and vesicular stomatitis virus with a green fluorescent protein (VSV-GFP) were cultured in our laboratory. MDCK and F81 cells inoculated with VSV-GFP showed obvious CPE. Conventional isolation and culture of mink enteritis virus, canine parvovirus, and raccoon dog parvovirus were performed using F81 cells because canine kidney cells do not produce CPE when inoculated with these viruses. Moreover, compared with dogs, cats and minks are more closely related, and the use of F81 cells reduces the effect of species specificity on the antiviral activity assay.

E. coli BL21 (DE3) competent cells were purchased from TransGen Biotech Co. Ltd. (Beijing, China). Six 6-month-old minks (*Mustela vison*) were purchased from a fur animal farm in Jilin Province, China. The minks were not vaccinated.

The animal experiments in this study were approved by Specialties, Chinese Academy of Agricultural Sciences (Beijing, China). This study was also approved (NO.ISAPSAEC-2021-24M) by the Laboratory Animal Management and Welfare Ethics Committee of the Institute of Special Economic Animal and Plant Sciences and the Chinese Academy of Agricultural Sciences, and all sampling procedures complied with the Institutional Animal Care and Use (IACUS) guidelines regarding the care and use of animals for scientific purposes.

### IFN-ε-encoding gene and ISG (ISG15, Mx1, and 2′-5′OAS) amplification

Mink peripheral blood lymphocyte collection, total RNA extraction, and complementary DNA (cDNA) production were performed as previously published (28). MiIFN-ε, ISG15, Mx1, and 2′-5′OAS1 cDNAs were amplified using specific primers (Table 1). SYBR-green quantitative reverse transcription PCR (qRT-PCR) was used to establish IFN-ε and ISG (ISG15, Mx1, and 2′-5′OAS1) expression and investigate their distribution in mink viscera, tissues, and organs.

**Table 1:** Sequences of primers used in this study.

**Cytokine**	**Predicted size (bp)**	**Primers (5^′^-3^′^)**
ORF	564	G CATTCGACCTTCACCATGA
		GAACAGACACCGGTTGATTT
SYBR real-time PCR	143	CTGCAGACCTTCAACCTCTTC
		CTAGGGTTCCCACCACTCAAG
*GAPDH*	102	GTCCCCACCCCCAATGTATC
		TCCCTCCGATGCCTGCT
*ISG15*	80	TCAGGGCTATCTGCTGCTTC
		TAGACCTCCCTGGCATCACC
*Mx1*	230	GTCCACCAGGTCAGGCTTTG
		TGCCACCACAGTATGCCC
*OAS1*	232	TGGAGCTCCTGACCGTCT
		TCAGCAGGGTCCAGAATCAC

ORF, open reading frame; PCR, polymerase chain reaction.

PCR amplification conditions were as follows: 95°C for 5 min, 94°C for 45 s, 53°C for 30 s, and 72°C for 50 s for 33 cycles. After amplification, 6 μL of the reaction was collected and separated *via* 1.5% agarose gel electrophoresis. The resulting PCR products were sequenced at Sangon Biotech Co. Ltd. (Shanghai, China). The SYBR green real-time reaction system (20 μL) comprised sterile water (6 μL), 2 × SYBR-green Premix Ex Taq (10 μL), primers (1 μL, 10 pmol/μL), and cDNA (2 μL, 10–20 ng/μL). Reaction parameters were as follows: 95°C for 45 s, initial denaturation at 95°C for 6 s, and 60°C for 27 s for 40 cycles. The Ct value was recorded after the reaction.

After sequencing, MiIFN-ε open reading frames were aligned using the basic local alignment search tool with IFN-ε sequences of various species obtained from GenBank, and a multispecies phylogenetic tree based on the nucleotide sequences of various IFNs was constructed using DNAStar 5.0 software (DNAStar, Madison, WI, USA). The signal peptide sequence and glycosylation sites of MiIFN-ε were predicted using online SignalP 4.1 Server and NetNGlyc Servers (http://www.cbs.dtu.dk/services), respectively. The 3D structure was predicted using the online Swiss Model (https://swissmodel.expasy.org/interactive).

### MiIFN-ε tissue distribution

The artificially reared minks were anesthetized with ether and sacrificed. The brain, lung, heart, liver, spleen, kidney, muscle, small intestine, abomasum, testis, ovary, and uterus tissues from three female and three male minks were collected by cell freezing tube and immediately frozen in liquid nitrogen. Liquid nitrogen was added to the dry pan to grind the tissues into dry powder, and the total RNA was extracted using Trizol (Invitrogen, Carlsbad, CA, USA) and an isolation kit (TaKaRa, Shiga, Japan). cDNAs were synthesized using the PrimeScript RT reagent kit (TaKaRa) with gDNA Eraser Perfect Real-Time. The detected RNAs were mixed with TB Green Premix DimerEraser and specific primers for MiIFN-ε. The results were statistically analyzed using GraphPad Prism 8 (GraphPad Prism software Inc. San Diego, CA, USA) and SPSS Statistics 25 software (IBM, Armonk, NY, USA). One-factor analysis of variance (one-way) was used to assess the significance of differences between multiple groups of averages (K ≥ 3).

### Molecular chaperone co-expression system construction and protein expression

The codon-optimized mink mature IFN-ε (MiIFN-ε)-encoding gene with N-terminal SUMO3 was synthesized by Sangon Biotech Co., Ltd. *Nco*I and *Xho*I restriction sites were added upstream and downstream of the synthetic sequence, respectively. The PUC57-SUMO-MiIFN-ε synthetic plasmid and pET28a were digested with *Nco*I and *Xho*I and the respective target sequences were recovered. The SUMO-MiIFN-ε-encoding gene and pET28a linearized vector were incubated overnight at 16 °C. The ligation product was transformed into *E. coli* BL21 competent cells. The bacterial solution was then spread on a Luria-Bertani (LB) agar plate containing 50 mg/mL kanamycin and cultured overnight in an incubator at 37°C. Seven single clones were selected, added into 5 mL of LB liquid medium (50 mg/mL), and cultured in an oscillating incubator at 37°C for 12 h. The plasmids were extracted, identified *via* PCR and restriction enzyme digestion, and analyzed by sequencing.

Recombinant positive *E. coli* BL21 competent cells containing the pET28a-SUMO-MiIFN-ε plasmid with kanamycin resistance gene were prepared, and the molecular chaperone plasmid pTf16 with chloramphenicol resistance gene was transformed into these cells. Positive colonies were selected on an LB plate containing 30 μg/mL kanamycin and 25 μg/mL chloramphenicol. A recombinant strain named pET28a-SUMO-MiIFN-ε-Tf16 was inoculated in LB medium (1:100) containing kanamycin, chloramphenicol, and L-arabinose and cultured at 37°C, shaking at 200 rpm. When the optical density (OD_600nm_) of the cells reached 0.5–0.7, isopropylβ-d-thiogalactoside (final concentration: 0.5 mmol/L) was added, and they were further cultured at 25°C, shaking at 180 rpm, for 16 h. Simultaneously, a pET28a vector blank control, control without isopropylβ-d-thiogalactoside, and control without L-arabinose were prepared. The supernatant was collected, and the solubility of the recombinant protein pET28a-SUMO-MiIFN-ε-Tf16 was determined *via* sodium dodecyl sulfate-polyacrylamide gel electrophoresis (SDS-PAGE). The soluble protein was purified using a His-NTA-resin affinity chromatography kit. Finally, the soluble protein was obtained by elution with an elution buffer (20 mmol/L Tris-HCl, pH 7.4, 500 mmol/L imidazole). Western blotting of partially purified 6 × His-tagged IFN-ε was performed as described previously (29) to identify the expression of SUMO-MiIFN-ε. The purified product was dialyzed with a 5 kDa cut-off dialysis bag overnight at 4 °C. Simultaneously, SUMO protease (20 mg/μL) was added to the dialysate, and a small sample was collected for SDS-PAGE analysis. The protein concentration was determined using PIERCE^TM^ BCA Protein Assay Kit (Thermo Scientific™, Rockford, IL, USA).

### MiIFN-ε *in vitro* antiviral activity

The antiviral activity of recombinant MiIFN-ε was evaluated *in vitro* using VSV as described previously (28). The titer and antiviral activity of IFN-ε were determined using the cytopathic effect inhibition (CPEI) assay (30–32). The dilution that achieved a 50% cytopathogenic effect (CPEI_50_) determined the antiviral titer of IFN. Titers were converted to international units (IUs) using a reference standard. F81 cells were subcultured in minimum essential medium (MEM) containing 8% fetal calf serum at 37°C and 5% CO_2_ until fully confluent. The medium was then discarded, and cells were digested with 0.25% trypsin and passaged in MEM containing 3% fetal calf serum. The cell suspension was inoculated with VSV-GFP at a ratio of 1:10 to produce a cytopathic effect (CPE) > 80%. The supernatant was collected and stored at −80°C.

#### Determination of the 50% tissue culture infective dose (TCID_50_)

The cell suspension was transferred to a 96-well plate (100 μL/well) and cultured in a humidified incubator with 5% CO_2_ at 37°C in the presence of 100 mL of 10-fold diluted VSV-GFP. Each dilution was assayed in octuplicate. TCID_50_ was calculated using the Reed-Muench method (33, 34). The viral culture was stored at −80°C for analysis.

#### Antiviral activity determination

Recombinant freeze-dried canine IFN-α (2 × 10^6^ IU/mL) (Lot No. 20200503, Tianjin Ringpu Biotechnology Co., Ltd.; Airport Economic Zone Branch, China) was used as the standard. F81 cells (1 × 10^5^ cells) were seeded in 96-well plates and incubated in a humidified incubator containing 5% CO_2_ at 37°C until 70% confluency. The medium was removed, and 100 μL of four-fold-diluted MiIFN-ε was added to each well and the same dilution standard reference was set, including F81 cells and virus controls. Experiments were performed in quadruplicate. The culture medium was removed, and the cells were infected with 100 μL of 100 TCID_50_ VSV-GFP. Then, MEM (100 μL) with 2% fetal calf serum was added to the control and IFN-ε control wells and cultured in an incubator for 24-−48 h. When the CPE in virus control wells was > 85%, F81 feline kidney cells were observed under an Olympus inverted microscope (CKX53, Olympus, Tokyo, Japan) and stained with 0.5% crystal violet for 30 min at 20–30°C. The number of infected cells was measured using crystal violet staining.

The F81/VSV-GFP system was used to determine MiIFN-ε titer and antiviral activity. One hundred microliters of the four-fold-diluted purified protein was added to each well. The plates were incubated at 37°C for 12 h in 5% CO_2_, and 100 mL of 100 VSV-GFP (TCID_50_ = 8) was added to each well. When the CPE in the virus control wells was > 85%, the antiviral activity of purified MiIFN-ε and standards was calculated, the culture medium was removed, and each well was washed thrice with 200 μL of phosphate-buffered saline. F81 cells were stained with 0.5% crystal violet for 30 min. The 96-well plate was rinsed with sterile deionized water, and 100 mL of 30% acetic acid was added to each well. Absorbance was measured at 540 nm in a microplate reader.

### Expression levels of ISGs, Mx1, and 2′-5′OAS1 in F81 cells

F81 cells were inoculated in a six-well plate, and the cells were allowed to grow to 70% confluence. MiIFN-ε recombinant protein was added for 12 h, and VSV-GFP was added after. After 24 h of challenge, the supernatant was gently discarded, and the cells were scraped. The normal control cells that were not inoculated with MiIFN-ε and VSV-GFP were collected as group A, the cells that were inoculated with VSV-GFP were collected as group B, the cells that were only inoculated with MiIFN-ε were collected as group C, and the cells that were attacked by the virus after adding IFN for 12 h were collected as group D. Three replicates were used for each group. The scraped cells from all groups were centrifuged at 3,000 × g for 10 min. Total RNA was extracted and reverse transcribed into cDNA, and the expression of ISG15, Mx1, and 2′-5′OAS1 was detected *via* qRT-PCR. The SYBR green real-time reaction system (20 μL) and procedure were the same as those applied for IFN-ε described above. The measured data were recorded with X ± S, and SPSS 25.0 software was used for data analysis. A *t*-test analysis was performed to assess differences between the two groups.

## Results

### MiIFN-ε-encoding gene analysis

**Figure 1:**
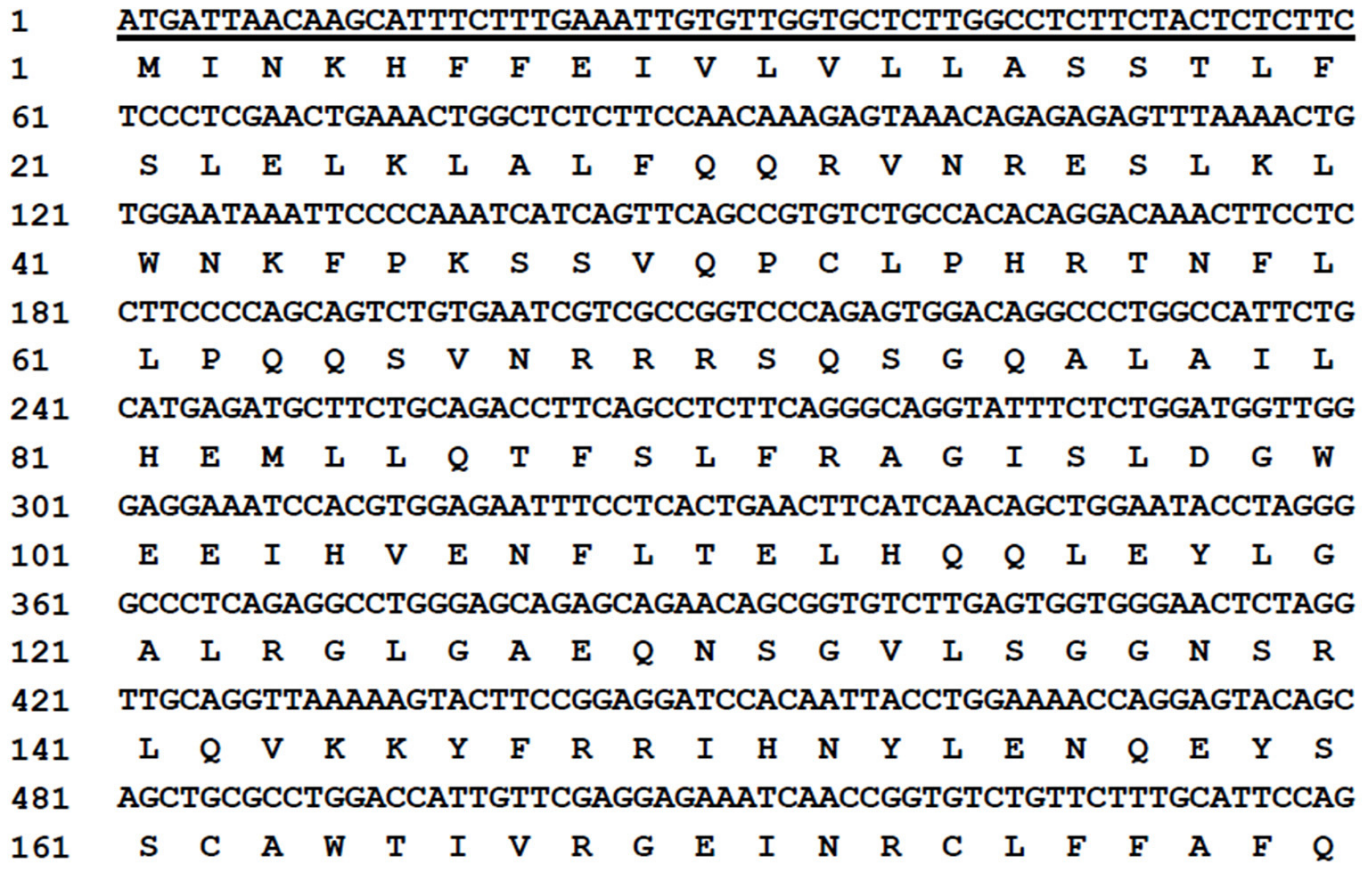
Nucleotide sequence and inferred amino acid sequences of the mink interferon (MiIFN)-ε-encoding gene. The amino acid sequences are presented under the nucleotide sequence. The underlined sequence represents the signal peptide gene coding region.

**Figure 2:**
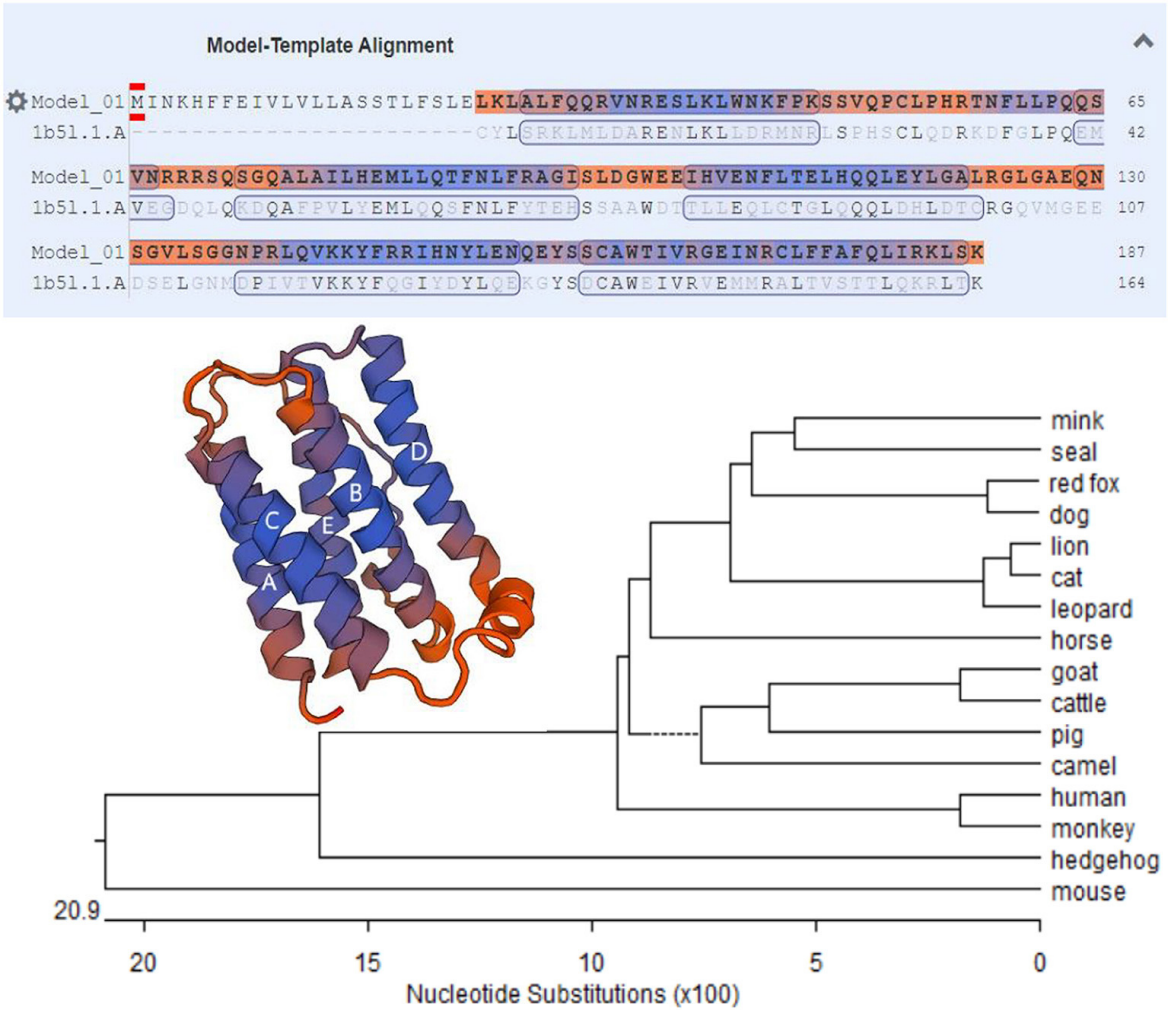
Phylogenetic tree of MiIFN-ε amino acid sequences of several mammal interferons (IFNs). The bar scale represents the genetic distance. The credibility value for each node is shown. Predicted three-dimensional structures of MiIFN-ε established using Swiss-Model 3D.

The MiIFN-ε-encoding gene fragment was obtained *via* qRT-PCR. A 564 bp gene fragment was confirmed using agarose gel electrophoresis. DNAStar analysis confirmed that the MiIFN-ε gene was 564 bp long and encoded 187 amino acids, containing 21 amino acid signal peptides, predicted using the online software SignalP 4.0 Server (Figure 1). The deduced MiIFN-ε gene encoded 167 amino acids, including three cysteine residues at positions 52, 162, and 174. There were no classical glycosylation sites (Asn-X-Ser/Thr). Sequence analysis showed that the homologies were 71.0–89.7% and 54.8–80.4% between MiIFN-ε and other species, including seal, fox, dog, lion, cat, leopard, horse, goat, cattle, pig, camel, human, monkey, hedgehog, and mouse. The MiIFN-ε gene was most similar to the seal IFN-ε gene, as the nucleotide homology and amino acid sequence homology reached 89.7 and 80.4%, respectively (Figure 2). Swiss-Model 3D and secondary structure prediction results showed that MiIFN-ε had five potential α helixes, similar to those of human and mouse IFN-ε. MiIFN-ε had one glycosylation site and five potential O-glycosylation sites, which might play important roles in protein folding, oligomerization, and stability, as reported in other studies (35). Evolutionary analysis showed that MiIFN-ε had the closest genetic relationship with seal IFN-ε, followed by canine IFN-ε (Figure 2).

### MiIFN-ε tissue distribution

**Figure 3:**
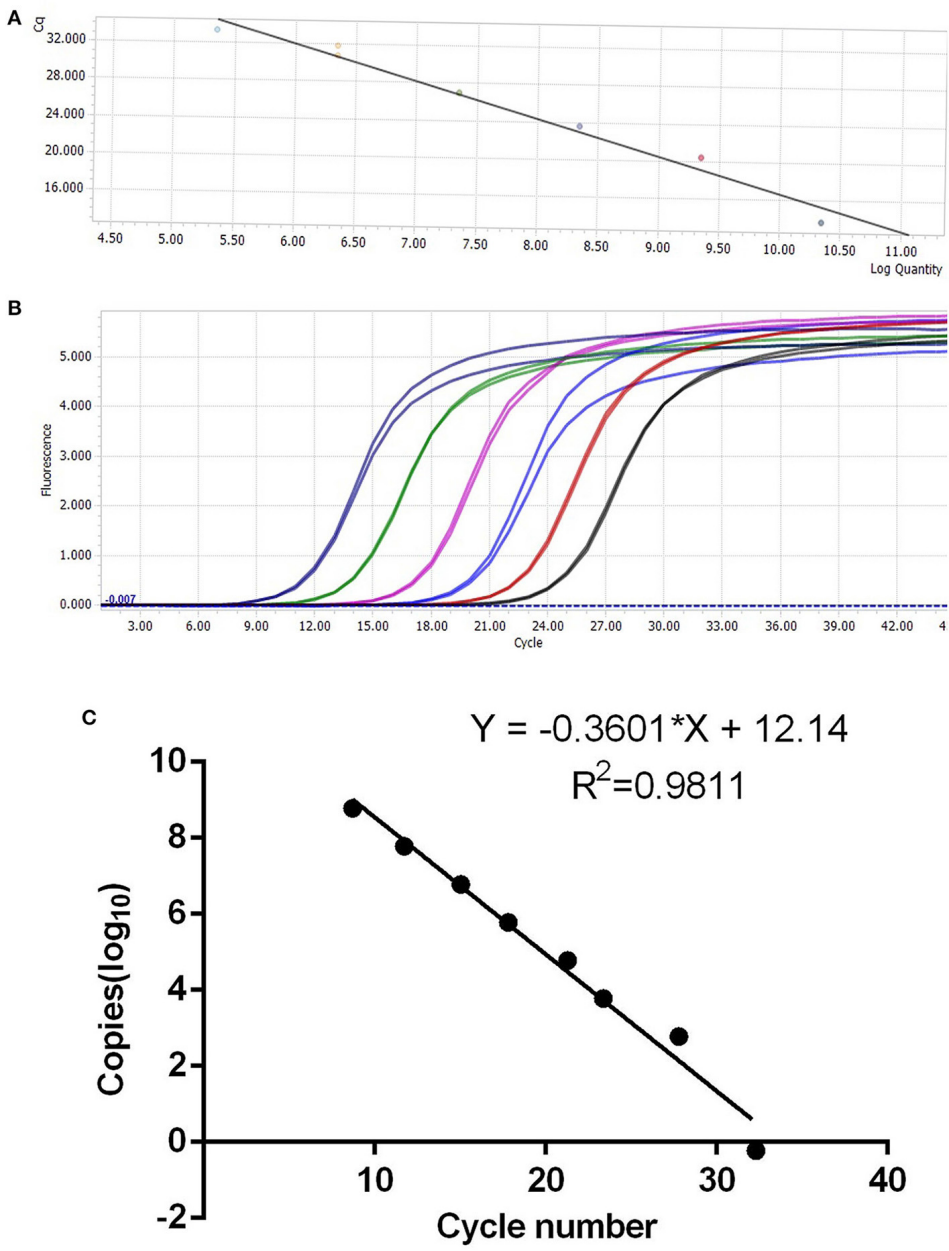
Establishment of IFN-ε SYBR fluorescence quantitative polymerase chain reaction. **(A,B)** Standard curve based on different dilution gradients of plasmids. **(C)** Standard curve for the correlation between copy number and cycle threshold value.

The synthesized cDNA was the template, and the target fragments of glyceraldehyde-3-phosphate dehydrogenase (254 bp) and IFN-ε (143 bp) gene sequences were obtained *via* PCR. The recombinant plasmid standard containing the target band was successfully constructed. After the concentration was determined, the copies were converted into 6.02 × 10^10^ copies/μL. The standard plasmid solutions containing 10^1^-10^7^ copies/μL were prepared *via* serial dilution using sterile water, and the OD_260nm_/OD_280nm_ was between 1.80 and 1.90 (Figure 3).

**Figure 4:**
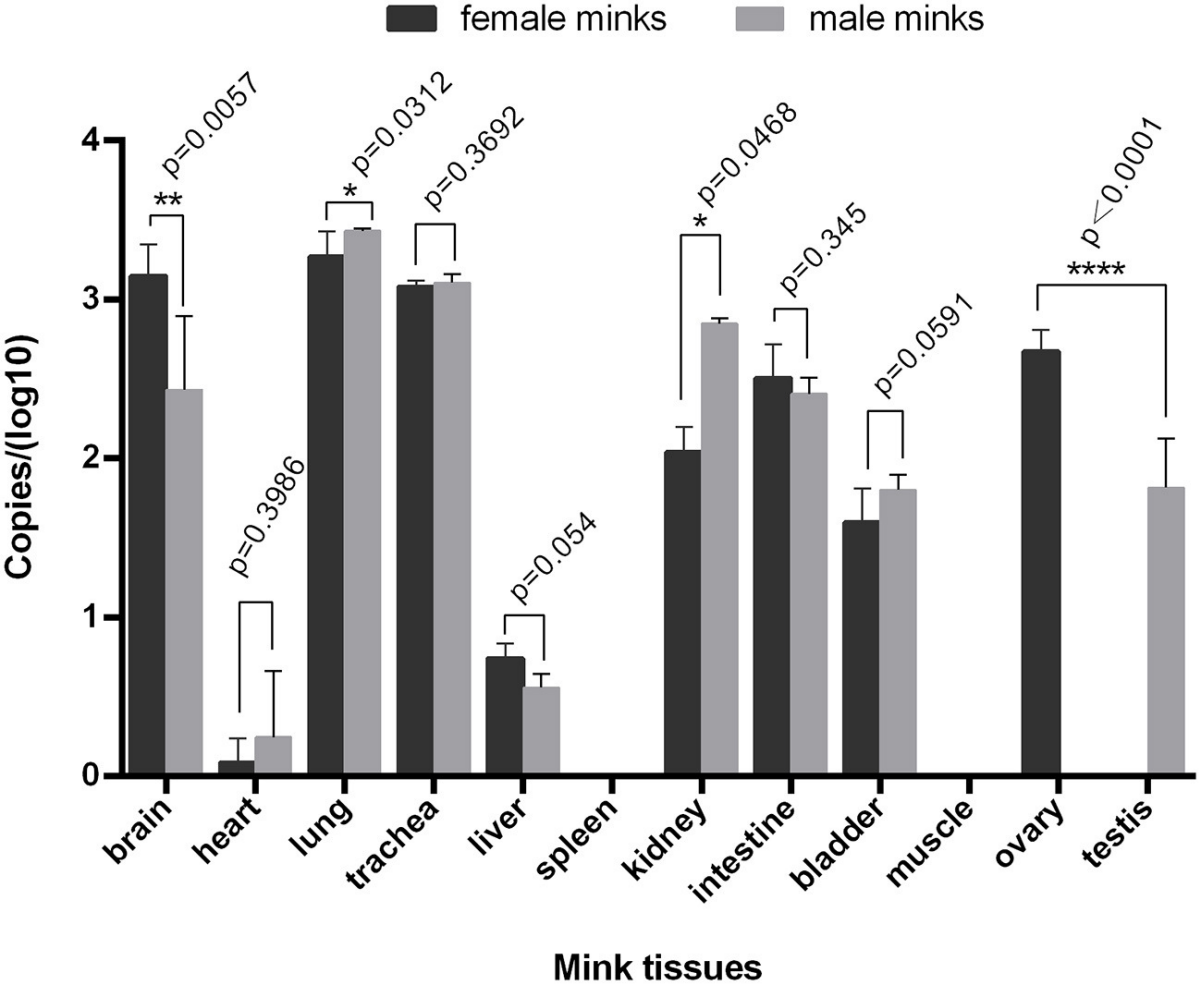
Distribution of IFN-ε in mink. The MiIFN-*f* gene was highly expressed in brain, lung, tracheal, kidney, intestinal, bladder, ovarian, and testis tissues; The expression of MiIFN-e displayed significant differences in female and male mink's brain (*p* < 0.01), lung (*p* < 0.05), reproductive organ tissue (*p* < 0.0001). *, *p* < 0.05; **, *p* < 0.01; ***, *p* < 0.001.

Fluorescence quantitative PCR showed that the MiIFN-ε gene was highly expressed in brain, lung, tracheal, kidney, intestinal, bladder, ovarian, and testis tissues; had lower expression in the heart and liver; and was not detected in spleen and muscle tissues (Figure 4). We found no significant differences in the trachea, intestine and bladder between female and male (p > 0.05). Moreover, the brain, lung, and kidney (0.01 < *p* < 0.05) reflected significant differences. Compared with male testis, female ovary showed higher expression levels and significant difference (*p* < 0.0001).

### MiIFN-ε expression and purification

**Figure 5:**
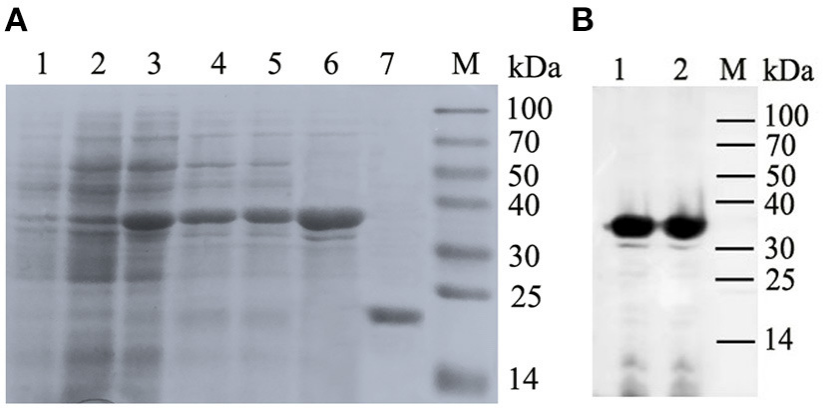
Co-expression of pET28-small ubiquitin-like modifier (SUMO)-IFN-ε and Tf16 chaperone plasmid, protein purification and Western blotting. **(A)** Expression of (SUMO)-IFN-ε and Tf16 chaperone plasmid and protein purification. Lanes: 1, Recombinant strain containing pET28a-SUMO-MiIFN-ε and Tf16 chaperone protein plasmid cultured; 2, cultured and induced for 48 h using L-arabinose; 3, Induced with isopropylβ-d-thiogalactoside for 48 h at 16 °C; 4, cell lysate supernatant; 5, centrifugal precipitation after cell lysis; 6, Elution buffer; 7, the purified SUMO-IFN-ε was digested using a SUMO protease to remove the SUMO label; M, protein molecular weight standard. **(B)** Western blot analysis of His-tag recombinant SUMO-MiIFN-ε by 6-poly histidine monoclonal antibodies. 1 and 2. SUMO-MiIFN-ε;M, protein molecular weight standard.

The 828 bp SUMO-MiIFN-ε ligation product contained 294 bp SUMO3 and 501 bp MiIFN-ε genes, encoding the target protein (31,749 Da). A ~32 kDa expressed protein was identified using SDS–PAGE (Figure 5A) and western blotting (Figure 5B). The concentration of the purified product was 0.6 mg/mL and it had a molecular weight of ~20 kDa after dialysis and SUMO protease digestion (Figure 5A).

### MiIFN-ε antiviral activity

**Figure 6:**
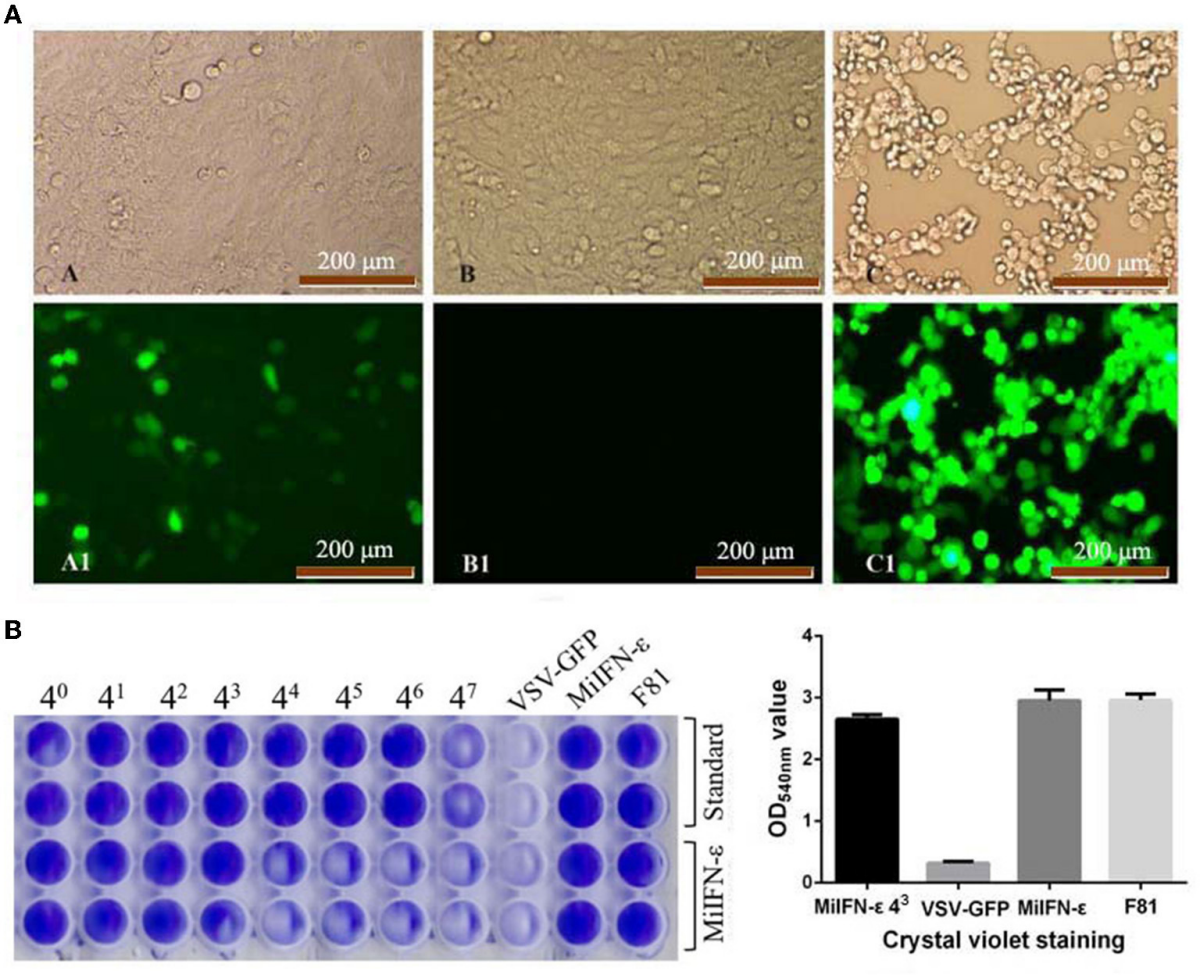
Fluorescence and crystal violet staining results of the inhibition of the replication of vesicular stomatitis virus tagged with green fluorescent protein (VSV-GFP) in Feline kidney 81 (F81) cells at 48 h. **(A)** A and A1, INF-ε 4^3^ Dilution; B and B1, Normal cell control; C and C1, VSV-GFP control. **(B)** Optical density at 540 nm after crystal violet staining.

IFN antiviral titer was taken as the maximum dilution of IFN per mL that could still protect half the cells from a viral attack. The antiviral titer of recombinant MiIFN-ε reached 4^3^. Protein concentration corresponding to the antiviral titer of MiIFN-ε was 413.5 pg/mL. At 4^3^ dilution, CPE was considerably reduced and a weak fluorescence signal was observed. Crystal violet staining rendered viable cells blue-violet, and non-colored dead cells were washed away with the effluent. The results were consistent with GFP fluorescence signal observation (Figure 6A). A significant difference at OD_540nm_ was observed between the IFN-ε 4^3^ dilution group and the VSV-GFP control group (*p* < 0.001). There was no difference among the IFN-ε 4^3^ dilution, IFN-ε, and F81 cell control groups (*p* > 0.05; Figure 6B).

The specific activity of recombinant IFN-ε was calculated using the following formula: international unit (IU/mL) = (sample CPEI_50_ dilution/standard CPEI_50_ dilution) × standard unit. The antiviral activity of recombinant IFN-ε was 1.3 × 10^4^ IU/mL.

### Effect of ISG expression levels in antiviral activity of MiIFN-ε

**Figure 7:**
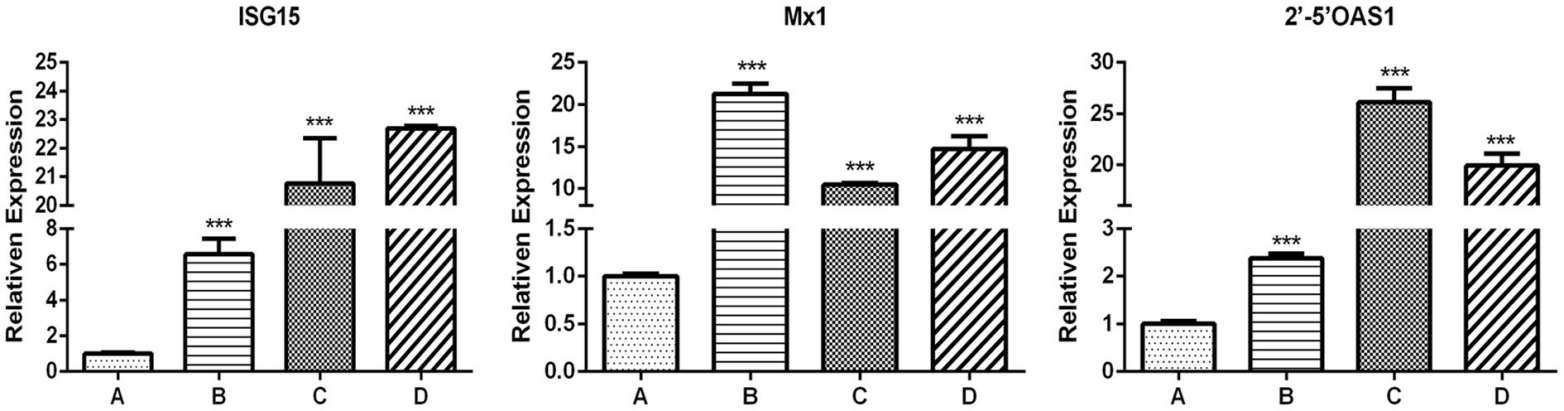
Expression of antiviral genes induced for 24 h by INF-ε in different F81 groups. **(A)** Cell control group. **(B)** VSV-GFP control group. **(C)** IFN-ε group. **(D)** MiIFN-ε-inhibited VSV-GFP group[[Inline Image]]. Compared with group A, the expression levels of ISG15, Mx1 and 2'-5'OAS in group B, C and D increased and displayed significant differences (*p* < 0.001), ^***^ indicates *p* < 0.001.

The results of ISG expression levels and analysis of differences are shown in Figure 7 and Table 2. After IFN-ε-treated cells were challenged with VSV-GFP, the expression of ISG in each group was considerably different, except for 2′-5′OAS1 expression between group A and group B. Compared with group A, the infected cells in groups B and C showed an increase in Mx1 expression, by 73 and 32 folds, respectively. ISG15 expression in groups B and C was over 26 folds higher than that in A. The expression of 2′-5′OAS1 in groups C and D was up to 15 folds higher than that in groups A and B. There was no significant difference in 2′-5′OAS1 expression between the F81 control group and the VSV-GFP control group.

**Table 2:** Changes in interferon-stimulated genes expression levels between different treatment groups during the antiviral activity of mink interferon (MiIFN)-ε.

**Groups**	**Groups**	**Significance (** ***p*** **-value)**		
		**ISG15**	**Mx1**	**2^**′**^-5^**′**^OAS1**
Normal F81 (A)	VSV-GFP	*p* < 0.0001	*p* < 0.0001	*p* > 0.05 (*p* = 0.057)
	MiIFN-ε	*p* < 0.0001	*p* < 0.0001	*p* < 0.0001
	MiIFN-ε+VSV-GFP (D)	*p* < 0.0001	*p* < 0.0001	*p* < 0.0001
VSV-GFP (B)	MiIFN-ε	*p* < 0.0001	*p* < 0.0001	*p* < 0.0001
	MiIFN-ε+VSV-GFP	*p* < 0.0001	*p* < 0.0001	*p* < 0.0001
MiIFN-ε (C)	MiIFN-ε+VSV-GFP	*p* < 0.43	*p* < 0.0001	*p* < 0.0001

GFP, green fluorescent protein; F81, Feline kidney 81 epithelial cells; MiIFN, mink interferon; VSV, vesicular stomatitis virus.

## Discussion

Interferon (IFN)-ε, a newly discovered member of the type I IFN family, exerts antiviral effects, and is significantly better in regulating mucosal immunity and neuromodulation than other type I IFNs. However, its antiviral effects are weaker than those of IFN-α and IFN-β *in vitro* (36, 37). IFN-ε is being studied for the treatment of viral diseases including condyloma acuminatum, verruca vulgaris, laryngeal papilloma, cervical cancer and other diseases caused by human papilloma virus infection, female genitourinary infections and some tumors. It is also being investigated as an alternative to other IFN strategies that are associated with adverse reactions. Unfortunately, there are few reports on animal IFN-ε; hence, its properties and biological effects are not clear. Currently, only mouse, dog, bovine, and sheep IFN-ε have been studied, and the biological functions of mink IFN-ε (MiIFN-ε) has not been reported.

The mink farming industry is important to Northeast China's economy. Several viruses can cause infection in minks, such as canine distemper, mink enteritis, and Aleutian disease (38, 39). Mink viral enteritis is an acute infection in minks that is associated with intestinal diarrhea and has a high mortality rate, which causes huge losses to the mink breeding industry. MiIFN-ε is highly expressed in mucosal tissues, such as intestinal and bronchial tissues. Here, we demonstrated that MiIFN-ε exerts antiviral effects *in vitro* and induces high expression of interferon-stimulated genes. Our findings provide insight into the biological functions of IFN-ε and lay foundations for future research.

Recently, research on animal IFNs has been conducted, and the development of efficient genetically-engineered antiviral drugs has become the focus of disease prevention and control (40). Here, the MiIFN-ε-encoding gene was amplified, pET28-SUMO-MiIFN-ε was constructed, and MiIFN-ε was successfully expressed in *E. coli*. The expressed product was soluble. There were three cysteine and two glycosylation sites in the sequence encoding the mature peptide, which could form disulfide bridges and undergo glycosylation to form glycoproteins. Secondary structure prediction showed that MiIFN-ε had five potential α helixes, consistent with human, mouse, and bovine IFN-ε.

In different species, IFN-ε expression differs in various organs and tissues, but IFN-ε tends to be highly expressed in many tissues including the brain, coronary smooth muscle endothelial cells, microvascular endothelial cells, mucosal epithelial cells of the reproductive tract, and bronchial epithelial cells (Table 3) (10, 22, 23, 27, 36, 38–42). There were not significant differences in the trachea, intestine and bladder between female and male. But the brain, lung, and kidney (0.01 < p < 0.05) reflected significant differences. a significant difference was noted between female ovary and male testis, female ovary showed higher expression levels than male testis. We presumed that this difference may be caused by estrogen.

**Table 3:** IFN-ε tissue distribution in different species.

**Species**	**Tissue**														
	**Brain**	**Heart**	**Lung**	**Trachea**	**Thymus**	**Liver**	**Kidney**	**Spleen**	**Intestine**	**Mesenteric** **lymph nodes**	**Cervix/Uterus**	**Ovary**	**Testis**	**Skin**	**Muscle**
Human	+^*^	–	+^*^	+^*^	–	–	–	–	+^*^	/	+^*^	+^*^	–	–	–
Mice	+	+	+	–	–	–	–	–	–	–	+^*^	+^*^	–	–	–
Macaque	+	+	–	–	+	–	+	–	+	/	+	+	+	–	–
Porcine	/	/	/	/	/	/	/	+	+	+	/	–	+	+	–
Bovine	–	–	–	–	+	+	+	–	+	/	/	/	+	–	–
Sheep	+^*^	–	+^*^	–	–	+	–	–	–	/	+^*^	+^*^	+	+	–

+, IFN-ε expression detected; +^*^, IFN-ε high expression detected; –, IFN-ε expression not detected; /, expression is not within the test range.

Results of fluorescence and cytopathologic observation of recombinant IFN-ε treatment in F81 cells showed that viral replication was significantly inhibited, while qRT-PCR analysis of IFN-ε-treated cells revealed upregulation of ISG15, Mx1, and 2′-5′ OAS. This showed that expression levels of antiviral proteins were elevated, indicating the induction of an antiviral state. 2′-5′OAS1 is a type I IFN-induced, intracellular double-stranded RNA sensor that generates 2′-5′-oligoadenylate to activate ribonuclease L (RNase L) for antiviral defense (43). 2′-5′-oligoadenylate combines with RNase L to form a dimer, thereby activating RNase L in the latent state. The activated RNase L can cleave RNA, including viral mRNA, to block the synthesis of viral protein polypeptide chains, thereby inhibiting the synthesis of viral proteins. In this study, qRT-PCR revealed that IFN-ε combined with the type I IFN receptor activated the expression of 2′-5′OAS1, and indirectly inhibited the replication of VSV-GFP in F81 cells. The activation of 2′-5′ OAS expression in F81 cells was one of the main antiviral mechanisms of MiIFN-ε.

This study has some limitations. First, although we induced soluble IFN-ε using the co-expression system at different temperatures, most expressed proteins were still present in the form of inclusion bodies, which affects the recovery rate of IFN-ε. Second, MiIFN-ε antiviral activity was assessed in canine and feline cells. Given the species specificity of type I IFNs, the calculation of specific activity values is not generalizable. For example, for the antiviral evaluation, F81 cells were used to evaluate the antiviral activity of MiIFN-ε, but there was no way to evaluate the effect of type I IFN species specificity on antiviral activity. Finally, in this study, we did not compare whether artificial infection with viruses such as MEV or canine distemper virus (CDV) affects the expression of MiIFN-ε *in vivo* or *in vitro*. Moreover, we did not successfully isolate the Aleutian mink virus. In the future, we will carry out *in vitro* and *in vivo* antiviral experiments using recombinant IFN-epsilon against MEV and CDV, as well as the expression levels of the IFN-stimulated genes ISG15, OAS, and MX1.

To summarize, MiIFN-ε antiviral activity was determined using the CPEI method in F81 cells. Our findings lay the foundation for further studies on IFN-ε and the development of antiviral drugs.

## Data availability statement

The datasets presented in this study can be found in online repositories. The names of the repository/repositories and accession number(s) can be found in the article/Supplementary material.

## Ethics statement

This study was also approved (NO.ISAPSAEC-2021-24M) by the Laboratory Animal Management and Welfare Ethics Committee of the Institute of Special Economic Animal and Plant Sciences and the Chinese Academy of Agricultural Sciences, and all sampling procedures complied with the Institutional Animal Care and Use (IACUS) guidelines regarding the care and use of animals for scientific purposes. Written informed consent was obtained from the owners for the participation of their animals in this study.

## Author contributions

HZ, SLu, and XB conceived and designed the experiments, analyzed the data, and wrote the manuscript. DZo, SZ, HL, BH, HW, CW, and SLi performed the experiments. DZh performed the main content of the experiment. SZ and SLi carried out statistical analyses. SLu provided guidance and revised the manuscript. All authors have read and approved the final manuscript.

## Funding

This study was supported by the Science and Technology Innovation Project of Chinese Academy of Agricultural Sciences (No. CAAS-ASTIP-2016-ISASPS), the National Nature Science Foundation of China (NSFC, No. 32072943), Jilin Scientific and Technological Development Program (20210509057RQ), Key Laboratory for Prevention and Control of Avian Influenza and Other Major Poultry Diseases, Ministry of Agriculture and Rural Affairs, China, and Key Laboratory of Livestock Disease Prevention of Guangdong Province (YDWS202205).

## Conflict of interest

The authors declare that the research was conducted in the absence of any commercial or financial relationships that could be construed as a potential conflict of interest.

## Publisher's note

All claims expressed in this article are solely those of the authors and do not necessarily represent those of their affiliated organizations, or those of the publisher, the editors and the reviewers. Any product that may be evaluated in this article, or claim that may be made by its manufacturer, is not guaranteed or endorsed by the publisher.
