# Supplementary material for: Isolation and characterization of the mink interferon-epsilon gene and its antiviral activity
Source: Front Vet Sci. 2023 Jan 27;9:972433. doi: 10.3389/fvets.2022.972433 (PMC9915148; doi:10.3389/fvets.2022.972433)
Supplement: Supplementary file 1 [file Data_Sheet_1.docx]

Supplementary material

**Figure 1.** IFN-ε gene melting curve.

IFN-ε gene melting curve


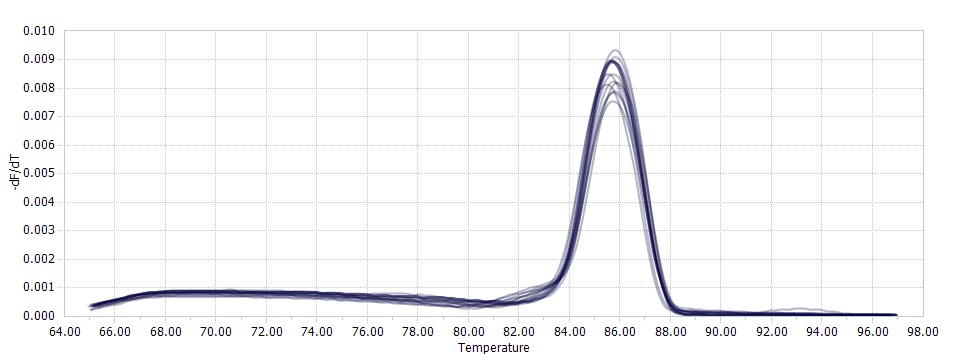


**Table 1.** The copies of mink IFN-ε in different tissues and organs by standard curve)

|  | Female 1# | | Female 2# | | Female 3# | | Male 1# | | Male 2# | | Male 3# | |
| --- | --- | --- | --- | --- | --- | --- | --- | --- | --- | --- | --- | --- |
| brain | 3.180712 | 3.213121 | 2.935844 | 2.910637 | 3.223924 | 3.429181 | 1.862746 | 2.190437 | 2.615355 | 2.651365 | 3.144702 | 2.122018 |
| heart | -0.258243 | -0.31946 | 0.483563 | 0.382735 | 0.101857 | 0.033438 | 0.735633 | 0.681618 | -0.22943 | -0.21143 | 0.296311 | 0.202685 |
| lung | 3.195116 | 3.493999 | 3.367964 | 3.295944 | 3.02947 | 3.238328 | 3.447186 | 3.42558 | 3.432782 | 3.414777 | 3.443585 | 3.429181 |
| trachea | 3.101490000 | 3.015066000 | 3.061879000 | 3.061879000 | 3.126697000 | 3.108692000 | 3.1771110 | 3.1663080 | 3.0906870 | 3.0798840 | 3.0654800 | 3.0510760 |
| liver | 0.858067000 | 0.804052000 | 0.753638000 | 0.771643000 | 0.602396000 | 0.656411000 | 0.4583560 | 0.4907650 | 0.6348050 | 0.6744160 | 0.5735880 | 0.5087700 |
| spleen | 0 | 0 | 0 | 0 | 0 | 0 | 0 | 0 | 0 | 0 | 0 | 0 |
| kidney | 1.898756 | 1.884352 | 2.255255 | 2.204841 | 2.028392 | 1.974377 | 2.817011 | 2.899834 | 2.867425 | 2.817011 | 2.824213 | 2.856622 |
| intestine | 2.219245 | 2.262457 | 2.582946 | 2.716183 | 2.604552 | 2.654966 | 2.302068 | 2.251654 | 2.456911 | 2.449709 | 2.500123 | 2.471315 |
| bladder | 1.412621 | 1.391015 | 1.772721 | 1.934766 | 1.549459 | 1.527853 | 1.938367 | 1.844741 | 1.66109 | 1.736711 | 1.833938 | 1.794327 |
| testis |  |  |  |  |  |  | 2.147225 | 2.16523 | 1.459434 | 1.470237 | 1.855544 | 1.790726 |
| ovary | 2.456911 | 2.622557 | 2.809809 | 2.824213 | 2.66937 | 2.651365 |  |  |  |  |  |  |
| muscle | 0 | 0 | 0 | 0 | 0 | 0 | 0 | 0 | 0 | 0 | 0 | 0 |

Table.2 Compared with F81 cell control group, the changes of interferon-stimulated genes (ISG15, Mx1 and 2’-5’OAS) in F81 cells treated with different ways.

| Gene | A | | | B | | | C | | | D | | |
| --- | --- | --- | --- | --- | --- | --- | --- | --- | --- | --- | --- | --- |
| ISG15 | 1.037660 | 1.053767 | 0.914536 | 7.292282 | 6.824933 | 5.586427 | 22.311320 | 19.155720 | 20.833230 | 22.721720 | 22.774280 | 22.599550 |
| Mx1 | 1.013977 | 0.970028 | 1.016688 | 19.91526 | 21.57604 | 22.31972 | 10.38849 | 10.33263 | 10.67232 | 14.078600 | 13.683080 | 16.448410 |
| 2’-5’OAS | 0.956068 | 0.982947 | 1.064097 | 2.485916 | 2.306971 | 2.357260 | 26.18093 | 27.46140 | 24.67347 | 21.04295 | 19.91288 | 18.73744 |

Fig.2 The melting curves of interferon-stimulated genes (ISG15, Mx1 and 2’-5’OAS)

HAPDH gene melting curve


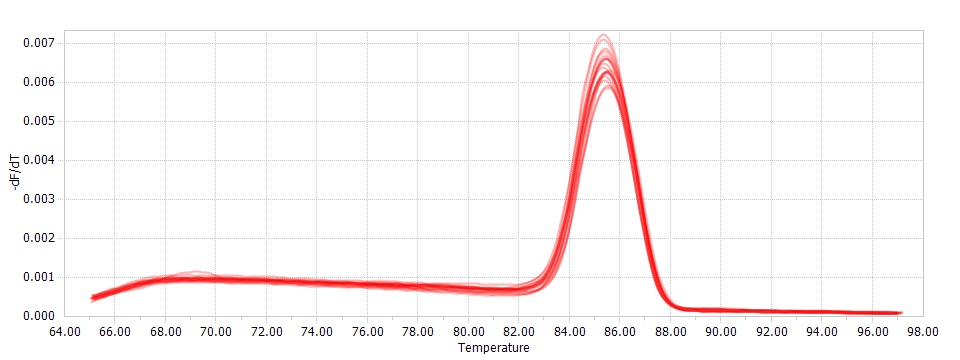

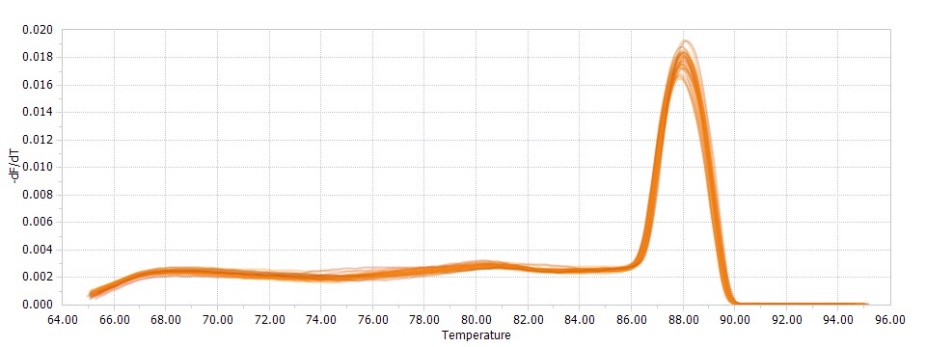


ISG15 gene melting curve

Mx1 gene melting curve


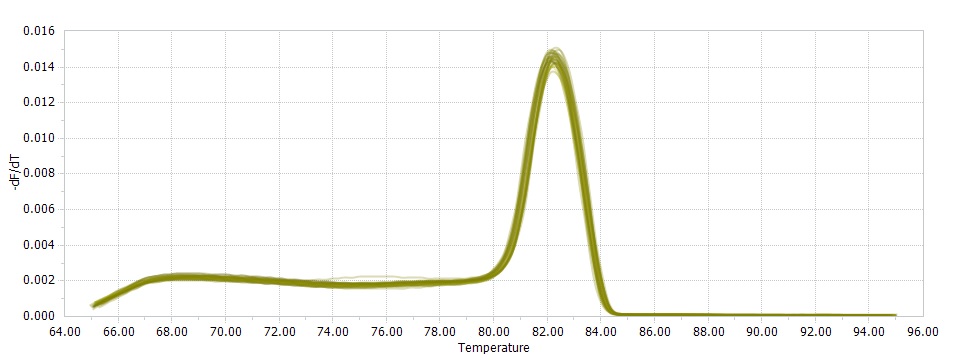

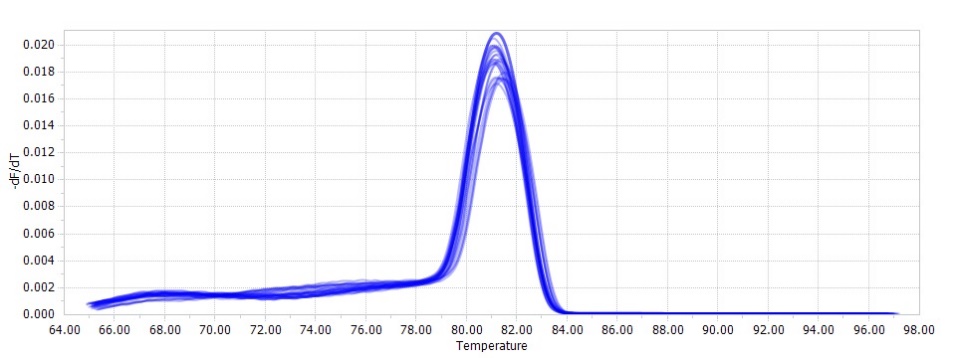


3’-5’ OAS gene melting curve
